# A Digital Infrastructure for Cardiovascular Patient Care Based on Mobile Health Data and Patient-Reported Outcomes: Concept Details of the Helios TeleWear Project Including Preliminary Experiences

**DOI:** 10.2196/41115

**Published:** 2023-03-03

**Authors:** Johannes Leiner, Sebastian König, Konstantinos Mouratis, Igor Kim, Pia Schmitz, Tanvi Joshi, Carolin Schanner, Lisa Wohlrab, Sven Hohenstein, Vincent Pellissier, Anne Nitsche, Ralf Kuhlen, Gerhard Hindricks, Andreas Bollmann

**Affiliations:** 1 Department of Electrophysiology Heart Center Leipzig University of Leipzig Leipzig Germany; 2 Real World Evidence and Health Technology Assessment Helios Health Institute Berlin Germany; 3 Helios Health GmbH Berlin Germany; 4 Helios Health Institute Berlin Germany

**Keywords:** mHealth, wearable, patient-reported outcomes, electrocardiogram, cardiovascular disease, atrial fibrillation, telemedicine, mobile health, telehealth

## Abstract

**Background:**

Mobile health (mHealth) approaches are already having a fundamental impact on clinical practice in cardiovascular medicine. A variety of different health apps and wearable devices for capturing health data such as electrocardiograms (ECGs) exist. However, most mHealth technologies focus on distinct variables without integrating patients’ quality of life, and the impact on clinical outcome measures of implementing those digital solutions into cardiovascular health care is still to be determined.

**Objective:**

Within this document, we describe the TeleWear project, which was recently initiated as an approach for contemporary patient management integrating mobile-collected health data and the standardized mHealth-guided measurement of patient-reported outcomes (PROs) in patients with cardiovascular disease.

**Methods:**

The specifically designed mobile app and clinical frontend form the central elements of our TeleWear infrastructure. Because of its flexible framework, the platform allows far-reaching customization with the possibility to add different mHealth data sources and respective questionnaires (patient-reported outcome measures).

**Results:**

With initial focus on patients with cardiac arrhythmias, a feasibility study is currently carried out to assess wearable-recorded ECG and PRO transmission and its evaluation by physicians using the TeleWear app and clinical frontend. First experiences made during the feasibility study yielded positive results and confirmed the platform’s functionality and usability.

**Conclusions:**

TeleWear represents a unique mHealth approach comprising PRO and mHealth data capturing. With the currently running TeleWear feasibility study, we aim to test and further develop the platform in a real-world setting. A randomized controlled trial including patients with atrial fibrillation that investigates PRO- and ECG-based clinical management based on the established TeleWear infrastructure will evaluate its clinical benefits. Widening the spectrum of health data collection and interpretation beyond the ECG and use of the TeleWear infrastructure in different patient subcohorts with focus on cardiovascular diseases are further milestones of the project with the ultimate goal to establish a comprehensive telemedical center entrenched by mHealth.

## Introduction

### Overview

In this paper, we describe the TeleWear project, which integrates mobile health (mHealth)–guided patient management by using patient-recorded health data and patient-reported outcome measures (PROMs).

Leveraging mHealth solutions to enhance integrated patient care has become a frequently pursued approach in recent years [1]. Application and benefits have been thoroughly studied among patients with cardiovascular diseases (CVDs) [2,3], which led to comprehensive position papers focusing on mHealth implementation in subspecialties of cardiovascular (CV) medicine, such as cardiac arrhythmia management [4,5] and CV prevention [6,7]. Patients with heart failure (HF) [8] and coronary artery disease [9,10] may also benefit from mHealth interventions; however, further research is needed as study results are in part inconsistent [11,12], and limitations with respect to patients’ digital capabilities exist [13]. Moreover, mHealth technologies are not yet implemented routinely in CVD patient care, mostly due to the lack of solid data [14].

Portable smart devices can be of great benefit for the detection of heart rhythm disorders [5,15]. Various technologies exist including wearables (eg, smartwatches), handheld electrocardiogram (ECG) devices, ECG patches, and smartphone apps [5]. Recent investigations revealed a good validity and reliability of wearable ECGs recorded by the Apple Watch (Apple Inc), including limb and precordial lead recording [16,17]. The usefulness of wearable technologies for atrial fibrillation (AF) screening and management of affected patients has been acknowledged by a novel European Society of Cardiology (ESC) consensus statement [5]. Nevertheless, randomized controlled trials (RCT) investigating prognostic benefits of mHealth interventions in AF management are scarce. The Chinese mobile Atrial Fibrillation App-II (mAFA-II) program leveraged a digital platform to enhance adherence to the ABC (“A”: Avoid stroke; “B”: Better symptom control; “C”: CV risk factors and comorbid conditions management) pathway for AF treatment [18] and showed positive results with regard to major adverse CV events [19-21]. The mAFA-II health app used by patients and doctors provided tools for supporting patient involvement, patient education, and follow-up as well as clinical decision support tools for physicians. The multicenter European TeleCheck-AF program was initiated as a response to the COVID-19 pandemic and included the establishment of a telemedical infrastructure accompanied by a smartphone app for remote AF patient management [22]. Rhythm monitoring within this smartphone app is achieved by photoplethysmography (PPG) sensors [5,22], a technology that is inferior to wearable ECGs in the detection of AF but has the advantage of widespread availability and simple application. First results of TeleCheck-AF showed good acceptance and satisfaction rates among patients and medical professionals [23,24]. Prognostic implications of this mHealth-guided telemedical infrastructure will be evaluated in an upcoming RCT [23].

Current ESC guidelines for AF treatment recommend routine collection of patient-reported outcomes (PROs) to assess patients’ quality of life especially in the light of therapeutic interventions [18]. Multidisciplinary expert groups proposed a set of validated tools for standardized PRO assessment (PROMs) in patient cohorts with AF [25,26]. However, PROMs are often not collected in clinical trials investigating patients with AF [27]. Furthermore, routine use of PROMs in clinical practice would be desirable, but implementation faces several obstacles, for example, with respect to data collection, analysis, interpretation, and logistics [28].

Herein, we aim to (1) introduce the TeleWear platform, (2) provide an overview of our initial feasibility study, and (3) summarize the planned TeleWear-AF study.

### The TeleWear Platform

**Figure 1:**
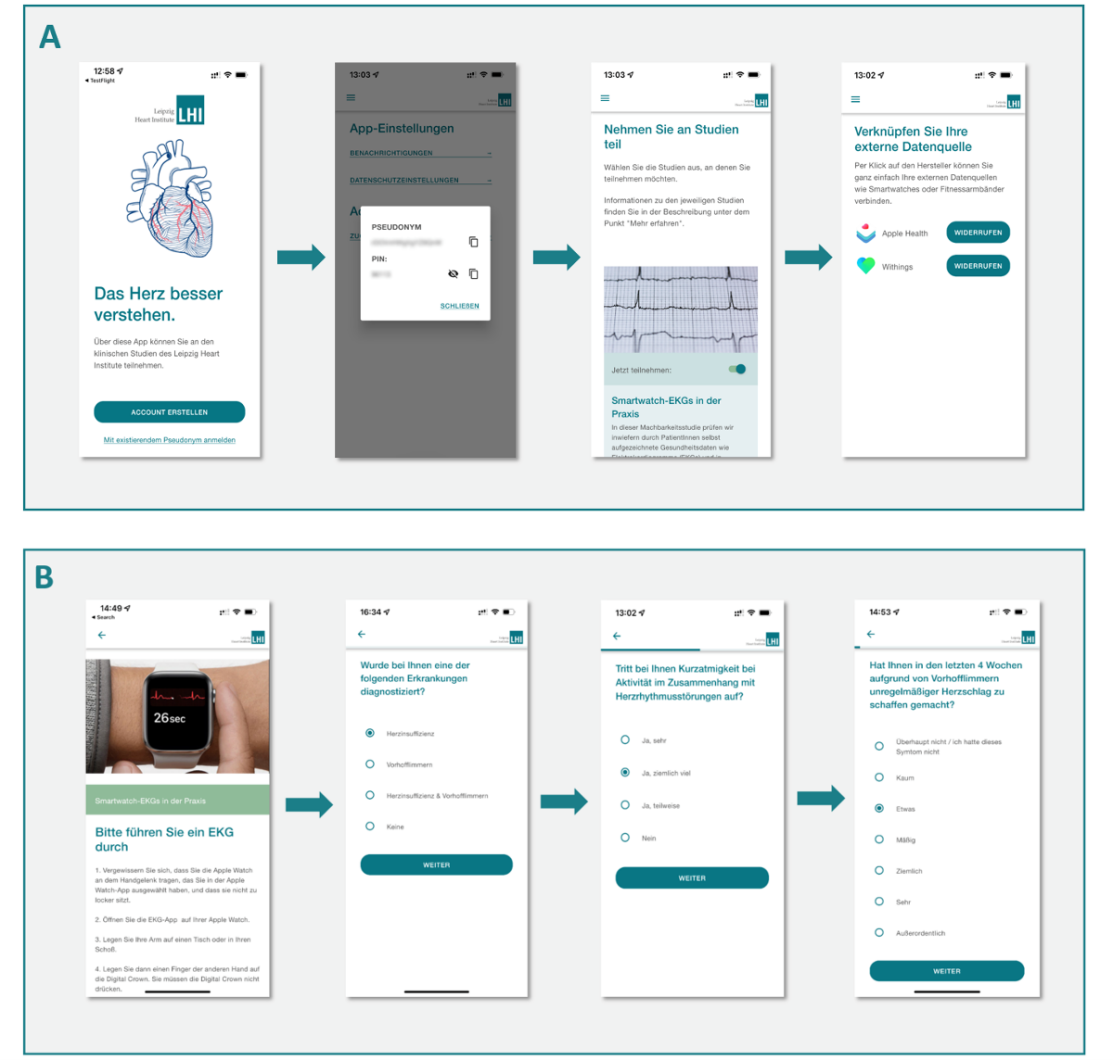
TeleWear app. (A) Screenshots from the original TeleWear app in German language. To participate in the TeleWear feasibility study, users create a personal account. The app automatically creates a pseudonym and personal identification number. The user has to allow linkage of data sources that communicate with the TeleWear app (eg, Apple Health). (B) Screenshots from the original TeleWear app in German language. Users can provide electrocardiograms and answer questionnaires via the app. The choice of questionnaires is dependent on the user’s medical history (presence of atrial fibrillation or heart failure).

**Figure 2:**
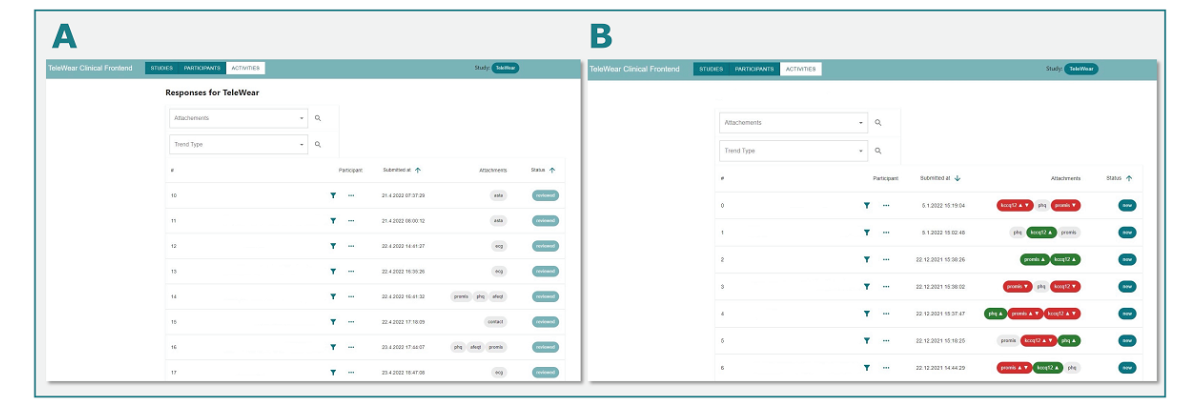
TeleWear clinical frontend. (A) Overview of activities by study participants. (B) Improvement and worsening of patient-reported outcome measure scores displayed in the clinical frontend.

**Figure 3:**
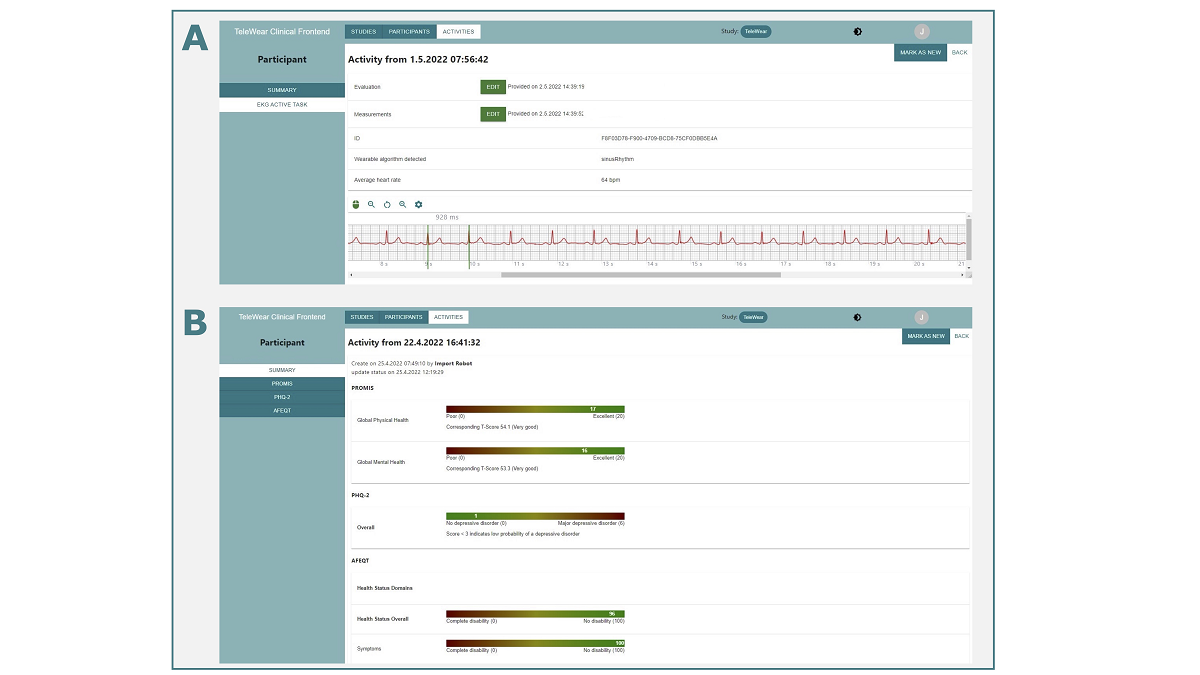
TeleWear clinical frontend - evaluation tools. (A) Electrocardiogram (ECG) evaluation tool for wearable-recorded ECGs in the clinical frontend. (B) Patient-reported outcome measure score viewer.

**Figure 4:**
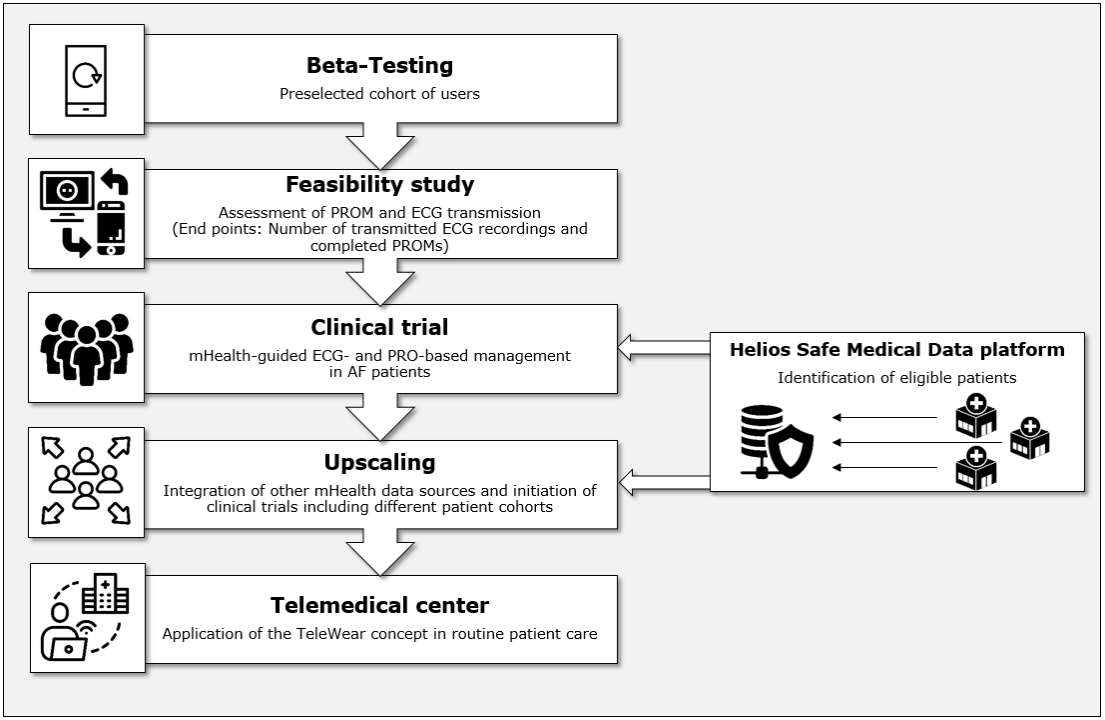
Phases of the TeleWear project. Currently, the TeleWear feasibility study is conducted, followed by a multicenter randomized controlled trial including patients with AF. For further explanations, see text. AF: atrial fibrillation; ECG: electrocardiogram; mHealth: mobile health; PRO: patient-reported outcome; PROM: patient-reported outcome measure.

With the TeleWear approach, we aim to develop, implement, and study an infrastructure for remote patient management that is based on 2 key elements: mobile-collected health data and PROs. Patients use a smartphone app that acts as a transmission platform and can integrate data from various health apps on the patient’s private smartphone, such as the smartphone’s built-in health data apps as well as apps for ECG recording with an accompanying wearable (Figure 1A). Furthermore, patients are asked to answer distinct questionnaires for PRO collection (Figure 1B). Collected data will then be transferred to a clinical frontend and reviewed by designated cardiologists using specific tools for evaluating recorded health data and PROM scores (Figures 2 and 3). The app is equipped with an application programming interface (API) that allows extensive customization; the integration of different data sources (health apps) and the addition of questionnaires are possible at any time. Because of this technical design, patients can provide comprehensive health data measurements to the clinical frontend. Currently, the project’s focus lies on patients with CVDs such as cardiac arrhythmias. However, a broad applicability in different areas of patient care is possible by using the existing infrastructure. In a first step, a feasibility study evaluates ECG and PROM transmission using wearables accompanied by a smartphone app in a real-world setting. Clinical applicability and effects of mHealth-guided PRO- and ECG-based patient management will then be assessed by conducting an RCT including patients with AF of the Helios hospital network. Further development includes the integration of other mHealth data beyond ECGs and evaluation of our approach in different patient cohorts with CVDs by conducting clinical trials, ultimately resulting in the establishment of a comprehensive mHealth-supported telemedical center (TMC; Figure 4).

## Methods

### Rationale and Design of the TeleWear Feasibility Study

The TeleWear project started with the realization of a pilot study initiated by the Leipzig Heart Institute (Leipzig site of the Helios Health Institute, Leipzig, Germany) that accompanied the platform’s development process and furthermore aims to assess the feasibility of wearable-recorded ECG and PROM capturing and its transmission to a health care facility. The Helios Health Institute constitutes a research institution adjacent to Helios Health GmbH, a large European health care provider, which includes the Helios hospital network consisting of 89 acute care hospitals in Germany. As has been shown previously [3,13], there is considerable restraint among patients with CVD to use health-related mobile technology. Therefore, the initial evaluation of user or patient satisfaction and engagement in the specific tool is a crucial part of implementing novel mHealth solutions. The study is led by physicians of the Department of Electrophysiology at the Heart Center Leipzig (University of Leipzig, Leipzig, Germany). Study participation is possible for every user of a wearable with ECG functionality and a compatible smartphone and age ≥18 years. The primary end point is the number of transmitted ECG recordings. Secondary end points, among others, include the number of completed PRO questionnaires, PROM and ECG evaluations by study physicians, symptom-rhythm correlation (ECG-PROM), type of response to the user and way of making contact, and user satisfaction with the app. TeleWear is co-financed with tax funds based on the budget passed by the Saxon state parliament via the Sächsische Aufbaubank.

### The TeleWear-AF RCT

**Figure 5:**
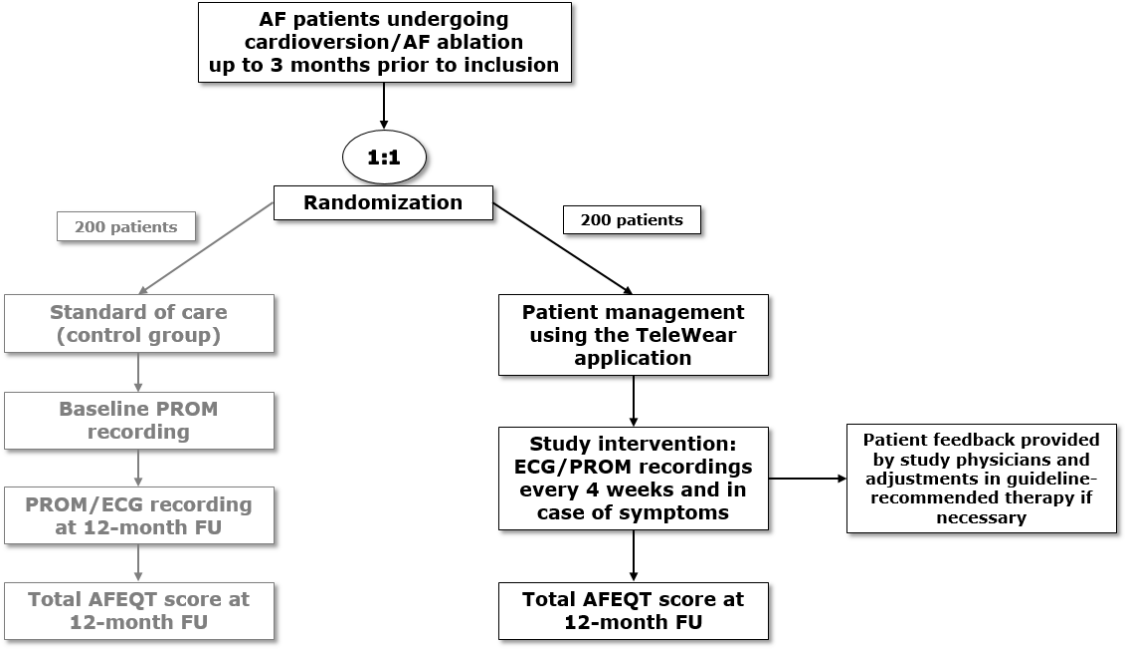
TeleWear-AF flowchart. This flowchart depicts the planned sequence of the TeleWear-AF randomized controlled trial. For further explanations, see text. AF: atrial fibrillation; AFEQT: Atrial Fibrillation Effect on QualiTy-of-life Questionnaire; ECG: electrocardiogram; FU: follow-up; PROM: patient-reported outcome measure.

With the feasibility study described above, the TeleWear infrastructure will be set up in a real-world environment and forms the basis for the initiation of an RCT that includes patients with AF of the Helios hospital network: TeleWear-AF. A study flowchart is provided in Figure 5.

#### Study Design

TeleWear-AF is a multicenter, interventional, open-label, parallel-group randomized controlled trial with superiority design.

#### Setting

Participants will be recruited from 10 tertiary hospitals in different cities in Germany. The study cohort will include patients with AF after cardioversion or catheter ablation for AF within the preceding 3 months of study enrollment. The total number of patients with AF treated in the respective study centers is approximately 20,500 per year with 2000 performed catheter ablations and 4000 cardioversions.

#### Participant Recruitment

Participant recruitment will be carried out (1) before AF-related interventions by clinicians in the participating hospitals and (2) via the Helios Safe Medical Data platform (HeSaMeDa). HeSaMeDa is designed to prospectively merge administrative data and data of electronic medical records from different Helios hospitals after obtaining a broad patient consent individually provided at hospital admission. In addition to consent to data donation, every patient treated in a specific Helios hospital can agree to be contacted for clinical research purposes. This platform will serve as a tool to identify suitable patients, contact them, and establish a multicenter study cohort. Inclusion criteria are as follows: age of at least 18 years, diagnosis of AF and cardioversion or catheter ablation for AF within the preceding 3 months of study enrollment, the possession of a smartphone and a device capable of ECG recording, and the ability to understand study requirements as well as self-reported basic digital literacy in a sense that patients feel comfortable of interacting with a mobile app.

#### Randomization and Allocation Concealment

After giving written informed consent, randomization will be performed using an electronic randomization tool in a 1:1 ratio to either standard of care (control group) or mHealth-guided PROM- and ECG-based management (intervention group). There will be no blinding of group allocation due to the nature of the intervention.

#### Intervention Group

The primary PRO tool used in this study is the Atrial Fibrillation Effect on QualiTy-of-life Questionnaire (AFEQT). It is a measure that contains 20 items resulting in 4 domains: symptoms, daily activities, treatment concerns, and treatment satisfaction. Each item is rated on a 1-7 numerical scale. An overall AFEQT score can be calculated with the 3 domains: symptoms, daily activities, and treatment concerns [29]. The recall period of AFEQT is 4 weeks [30], and it can usually be conducted in 5-10 minutes. Other PROMs used in this study include Patient Reported Outcomes Measurement Information System (PROMIS) Global Health and Patient Health Questionnaire-2 (PHQ-2). In the interventional study arm, patients will provide PROMs and ECGs every 2 weeks and in case of symptoms via the TeleWear app. In case of missing data from routine 2-week intervals, up to 3 electronic reminders will be sent to the participants via the app. In addition, telephonic reminders are possible if patients do not respond to electronic reminders. These data form the basis for adjustments in guideline-recommended therapeutic measures [18]. Predefined triggers (eg, AF detection on ECG and worsening of symptoms with PROM-specific thresholds) will lead to certain therapeutic interventions (eg, repeat cardioversion, medication adjustments, reablation, etc.).

#### Control Group

Patients randomized to the control group will get access to the app but will only be able to provide PROMs at baseline and 12-month follow-up as well as a final study ECG after 12 months from enrollment (standard of care). Patients will be informed that data are only collected for scientific research purposes, no alerts are generated, and no feedback by study physicians is provided. Patients’ symptom and rhythm management will follow the current standard of care.

#### Primary and Secondary End Points

The primary end point will be the total AFEQT score at 12 months from enrollment. Secondary end points include the PROMIS Global Health scores, the PHQ-2 score, the proportion of patients with AF after 12 months from enrollment, the time to detection of arrhythmia recurrence after the index intervention (catheter ablation or cardioversion), the number of unplanned hospitalizations for CV reasons, the number of unplanned outpatient visits, and the health care burden defined as the number of AF-related interventions within 12 months from enrollment.

#### Research Hypothesis

Our research hypothesis is that a digital PROM- and ECG-based management of patients referred for rhythm control of AF will improve patients’ quality of life during follow-up compared with standard of care.

#### Sample Size Calculation

Considering the expected mean AFEQT score at 12 months in the control group and a clinically meaningful difference of at least 5 points, which is also consistent with the expected treatment effect based on previous studies [29,31-33], the required sample size would be 176 patients per group for testing the null hypothesis. Assuming a dropout rate of 10%, we plan to include 200 patients per study group. On the basis of the results of the beta-testing phase of TeleWear, we estimate a consent rate of 15%, and therefore the screening of 2666 patients is projected. The sample size calculation is based on the independent-sample Student *t* test with a 2-tailed *α* level of .05 for a power of 80%.

#### Other Data

The clinician workload, clinician system acceptability, and patient satisfaction of the app and interventions will be assessed through surveys and interviews. Demographics, clinical characteristics, follow-up information, and adverse events of the interventions will also be collected.

#### Data Management

Two trained research physicians will review the clinical frontend of the TeleWear platform on a regular basis (daily on business days). Results from PROMs, ECG evaluations, and clinical data from electronic medical records will be collected using an electronic case report form on a secured server within the Helios hospital information system. Data will be stored using a study-specific patient identifier to address data privacy requirements. All data will be quality checked regularly by a data monitor.

#### Data Analysis

Per-protocol analyses will be conducted. To be included in the analysis, a participant must provide AFEQT data and ECG data from baseline, 12-month follow-up, and at least 2 additional time points. If a participant meets the withdrawal criteria, no data will be included in the analysis. Two-sided *P* values of <.05 are considered to be statistically significant. Continuous variables will be presented as mean (SD) or median (IQR). Comparisons between groups will be conducted using the Student *t* test or the Wilcoxon rank-sum test. Categorical variables will be presented as frequencies or proportions and compared between groups using the chi-square test.

#### Ethics and Dissemination

Ethical approval for the TeleWear feasibility study was given by the Ethics Committee at the Medical Faculty of the University of Leipzig (AZ163/21-ek). Written informed consent is obtained electronically from every participant.

Regarding the TeleWear-AF RCT, all recruited patients will be required to give written informed consent. The study will comply with the principles of the Declaration of Helsinki, and any subsequent amendments to the protocol will be submitted for further review and approval. Study centers will gain approval from their hospital-specific ethics committees. The results of this study will be disseminated through peer-reviewed publications and academic conferences.

## Results

### Study Participation

Part of the feasibility study was the development of a health app that serves as a digital platform for study participation and transmission of ECGs and PROMs. The participation process is depicted in Figure 1A: after download, users can register within the app. To ensure security and data privacy, a pseudonym and personal identification number are created automatically. The user is informed about purpose and methods of the study and important data protection issues. Most importantly, the user is notified that the app does not constitute a medical device and cannot be used in case of an emergency. Consent for study participation is given electronically. The access to other apps on the user’s smartphone in which health data (eg, ECGs) are recorded and stored must be allowed explicitly.

Users can then provide ECGs and PROs at their convenience (Figure 1B). Multiple transmissions are possible at any time. The choice of PROMs is based on the user’s medical history: if the user has been diagnosed with AF or HF previously, the appropriate set of PROMs will be selected (see below). Study participants can choose if they would like feedback on the transmitted health data after an evaluation by study physicians took place and submit a contact form with their desired way of making contact (eg, email and telephone call). Finally, users can provide feedback on their satisfaction with the app.

The study was preceded by a beta-testing phase with preselected users to test the app’s usability and implement technical adaptions within the app or clinical frontend (Figure 4).

### Technical Aspects

**Figure 6:**
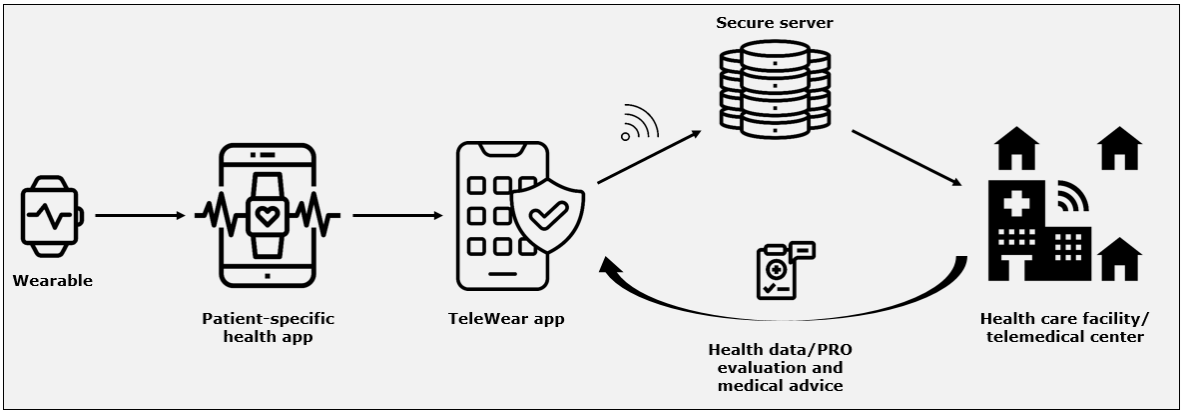
Data flow. Schematic illustration of the dataflow within the TeleWear infrastructure. Patients record health data (eg, electrocardiograms) with wearables that connect to a specific health app on the patients’ smartphone (eg, Apple Health). If data access is allowed, the TeleWear app collects these health data and transmits them to a health care facility or telemedical center (TMC) via a secure server connection together with patient-reported outcomes (PROs), where evaluation can take place. The patient receives medical advice by TMC physicians using the preferred communication channel (eg, telephone call, video consultation, direct messaging, or email).

The TeleWear app and the clinical frontend were developed using an existing API (Thryve mHealth Pioneers GmbH) that allows the integration of apps and corresponding wearables by various manufacturers. Through this approach, linkage of other health apps that record data such as blood pressure, activity, or oxygen saturation is possible at any time as is the addition of other questionnaires and PROMs. However, for the purpose of the feasibility study, only the transmission of ECGs and a predefined set of PROMs is implemented. Currently, the app allows data exchange with Apple Health (Apple Inc) and Withings Health Mate (Withings; Figure 1A). Integration of other apps is part of further development. ECGs (and health data) recorded by manufacturer-specific wearables are saved within the respective app and then transferred to the TeleWear app, which allows transmission to the Leipzig Heart Institute (Figure 6). ECGs are transmitted in .xml-file format, which can be viewed and analyzed within the clinical frontend (Figure 3A). PROMs are stored locally within the app. All data are transferred pseudonymized. The TeleWear app itself does not provide any medical advice or automatic ECG or PROM evaluation and only serves as a transmission tool. Therefore, the app does not constitute a medical device and requires no CE mark, which was confirmed by the responsible ethics committee (for the vote, see above). The required app is available for free from app stores on iOS and Android devices.

### Clinical Frontend

The clinical frontend was specifically developed for the TeleWear platform using the Next.js (Vercel Inc) web development framework. It can be accessed via every web browser within the Helios network with a personalized login. Login data is provided only for study personnel.

The TeleWear clinical frontend comprises an overview of all activities by study participants, an ECG evaluation tool to perform measurements, and a PROM score viewer (Figures 2 and 3). Those scores are calculated automatically based on instructions provided by the publishers of the respective PROMs. If one questionnaire is answered multiple times, changes in PROM scores (improvement or worsening) are displayed by the system so that physicians can assess potential changes in the participant’s health status (Figure 2B). Study personnel contact the user via the provided contact form directly within the clinical frontend if feedback is desired and provide the physician’s evaluation and recommendations for further proceedings.

### Patient-Reported Outcomes

Currently, 5 different tools to measure PROs are implemented in the TeleWear app. The choice of questionnaires for AF and HF is based on recommendations by the International Consortium for Health Outcomes Measurement [25,34], with one additional questionnaire addressing cardiac arrhythmias in general.

PROMIS Global Health provides 2 summary scores to assess patients’ general health perception [35]. The AFEQT was designed and validated specifically for patients with AF [30] as was the Kansas City Cardiomyopathy Questionnaire (KCCQ-12) [36] for patients with HF. The PHQ-2 represents an ultrashort screening instrument for depression [37]. Additionally, the Arrhythmia-Specific Questionnaire in Tachycardia (ASTA) was developed for patients with cardiac arrhythmias in general [38].

Depending on whether participants were previously diagnosed with AF or HF, the app redirects to either AFEQT (AF), KCCQ-12 (HF), or both. If neither AF nor HF is present, ASTA will be chosen. PROMIS and PHQ-2 are recorded in every participant.

### First Experiences

During the first 4 weeks since the initiation of our TeleWear feasibility study, 18 participants gave consent. In total, 134 ECGs were submitted by 18 users (range 1-86 ECGs), and 35 PROMs (3× ASTA, 11× PROMIS Global Health, 10× PHQ-2, and 11× AFEQT) completed by 12 users. The most common ECG finding was normal sinus rhythm, but findings also included AF, premature ventricular contractions, supraventricular premature beats, and first-degree atrioventricular block. Of 9 users who gave adequate feedback, 8 reported that the platform was user-friendly and easy to use, and PROMs were comprehensible.

## Discussion

### Principal Findings

Herein, we describe the cornerstones of the recently initiated TeleWear project and its future directions with the goal to develop a telemedical infrastructure supplemented by mHealth solutions, which incorporates an approach to patient care based on PROs and digital health data. However, possible benefits for patients need to be assessed in clinical trials before broad application in clinical practice is considered. To the best of our knowledge, the upcoming RCT (TeleWear-AF) described above will be the first to investigate PROM- and ECG-based management of patients with AF using mHealth solutions.

Previous experiences with mHealth approaches in AF patient management yielded promising results. In both mAFA-II and TeleCheck-AF, a designated smartphone app for recording symptoms, adverse events, changes in medication, and health data was required [22,39]. In contrast to our approach, heart rate and rhythm were evaluated by using PPG recordings with the advantage that no wearable in addition to the patient’s smartphone was needed. However, an AF diagnosis is not possible via PPG signals [18]. Moreover, both studies did not comprise structured PROM capturing. TeleWear can therefore add to existing data and provide real-world evidence on potential benefits of mHealth in AF management with focus on PROs and wearable ECGs. Of note, provision of educational material via the app is currently not planned in contrast to the previously mentioned studies, mAFA-II and TeleCheck-AF.

TeleWear can also serve as an example for clinical trial digitalization, which drew more attention in the light of the COVID-19 pandemic and should be of major importance in the near future. Not only patient recruitment and follow-up can be simplified through digitizing clinical studies but also significant advantages regarding data capturing and data analytics are to be expected [40]. As outlined before, the TeleWear platform can host multiple studies at the same time and can be tailored to the requirements of every trial that includes questionnaires and digital health data.

### Outlook

Future perspectives of the TeleWear project include integration of other health data collected by smart devices. Wearables are able to measure blood pressure without the need for additional cuffs; however, further clinical validation of available devices and corresponding algorithms is needed [41]. Physical activity that is continuously tracked by smart devices could be of prognostic relevance in patients with HF [42] and has been acknowledged as an important tool in this patient cohort [43] albeit the correlation between physical activity and objective measures of quality of life is not yet thoroughly studied [42]. Furthermore, sleep duration has been proposed as a predictor of CVD [44] and can be measured with wearables [45]. Besides widening the spectrum of health data collection, the use of the TeleWear infrastructure in different patient subcohorts with focus on CVD will be a further milestone of the project. However, TeleWear represents a flexible platform, which is not necessarily limited to specific disease entities such as CVD, and application in other areas of patient care is conceivable. On the basis of the data derived from clinical trials and our gained experience, we ultimately aim to establish a TMC using the TeleWear framework that simplifies and improves routine patient care (Figure 4).

Artificial intelligence techniques such as deep learning have the potential to revolutionize ECG analysis and interpretation. Investigations studying deep learning–based predictions of cardiac and also noncardiac diseases using ECG tracings yielded impressive first results [46]. We aim to integrate machine learning or deep learning–based ECG analysis tools as part of the platform’s future development but with preceding careful consideration of ethical and legal aspects.

### Limitations

Despite the multiple advantages and possibilities of mHealth, some obstacles in implementation could arise with respect to the digital capabilities of distinct patient cohorts. A survey conducted recently in a cohort of patients with HF showed that less than half were interested in a digital solution for PROM capturing in the context of an HF registry and many participants preferred a web-based solution over an app-based solution [13]. Possible differences in digital capabilities may apply to patients with AF, as first TeleCheck-AF results showed good acceptance of the digital solution in an AF cohort with a median age of 64 years [23]. However, it is of crucial importance to consider digital literacy first when offering mHealth solutions to respective patients [5]. It must be noted that one important limitation of digital health app is the availability of devices among people with limited financial resources due to costs associated with, for example, smartphones and high-speed internet [47]. The underrepresentation of specific patient subgroups is even more exacerbated when wearables in addition to smartphones are required. Moreover, because of data privacy considerations, several patients might refrain from using mHealth [47]. Hence, providing a secure platform for patients and users was one of the central goals during the TeleWear app development process (Figure 1A). In summary, there are important limitations to all mHealth solutions in terms of the patients’ ability and willingness to use them, which also leads to the exclusion of distinct subgroups in clinical trials.

To conclude, one great future challenge when it comes to the implementation of digital health tools in clinical practice will be the adaption of reimbursement policies [47]. Creating a robust body of evidence for mHealth is the most important step toward possible advances, and TeleWear will hopefully contribute to this regard.

### Conclusions

TeleWear represents an mHealth approach comprising PRO and mobile-collected health data capturing. During the project’s pilot phase, we are exploring the feasibility of ECG and PROM transmission, subsequent evaluation by a health care facility, and the feedback loop back to the user. PRO- and ECG-based patient management using the TeleWear app will then be evaluated in a cohort of patients with AF. Integration of other mHealth data sources and clinical application of our TeleWear approach in different CVD subcohorts are further steps toward the development of a TMC guided by mHealth technologies.

## Abbreviations

AF: atrial fibrillation
AFEQT: Atrial Fibrillation Effect on QualiTy-of-life Questionnaire
API: application programming interface
ASTA: Arrhythmia-Specific Questionnaire in Tachycardia
CV: cardiovascular
CVD: cardiovascular disease
ECG: electrocardiogram
ESC: European Society of Cardiology
HeSaMeDa: Helios Safe Medical Data Platform
HF: heart failure
KCCQ-12: Kansas City Cardiomyopathy Questionnaire
mAFA-II: mobile Atrial Fibrillation App-II
mHealth: mobile health
PHQ-2: Patient Health Questionnaire-2
PPG: photoplethysmography
PRO: patient-reported outcome
PROM: patient-reported outcome measure
PROMIS: Patient-Reported Outcomes Measurement Information System
RCT: randomized controlled trial
TMC: telemedical center

## Data Availability

Helios Health and Helios Hospitals have strict rules regarding data sharing because of the fact that health data are a sensible data source and have ethical restrictions imposed because of concerns regarding privacy. Data sets generated during the current feasibility study and later the TeleWear-AF RCT are available on reasonable request from the Leipzig Heart Institute at Helios Health Institute [48]. Please direct queries to the data protection officer (email: info@leipzig-heart.de) and refer to study “TeleWear.”
